# Can Adults Accurately Judge Child Weight Status?

**DOI:** 10.3390/children12070836

**Published:** 2025-06-25

**Authors:** Bethany J. Ridley, Kristofor McCarty, Robin S. S. Kramer, Martin J. Tovée, Piers L. Cornelissen

**Affiliations:** 1 Department of Psychology, Northumbria University, Newcastle upon Tyne NE1 8ST, UK; brgamble@arden.ac.uk (B.J.R.); kristofor.mccarty3@northumbria.ac.uk (K.M.); piers.cornelissen@northumbria.ac.uk (P.L.C.); 2 School of Psychology, University of Lincoln, Lincoln LN6 7TS, UK; rkramer@lincoln.ac.uk

**Keywords:** childhood weight, overweight, parents, BMI categories

## Abstract

Background/objectives: This study addresses two questions: what body sizes/shapes do participants believe correspond to the boundaries of the National Child Measurement Programme (NCMP) weight categories for children aged 4–5 and 10–11 years old, and are these judgements altered by using terminology encouraging positive action by parents? Methods: The study used photorealistic computer-generated stimuli based on 388 3D scans of children in a method of adjustment task. We first asked participants to estimate the boundaries between weight status categories as described by the NCMP. To test validity, we asked a second set of participants to estimate the body that represented exemplars of each weight category (the exemplars should fall between the boundary estimates). We then recruited a third set of participants to determine whether substituting positive action terminology for the weight status definitions altered the boundary positions. Results: First, validity was confirmed. Second, we found a compressed response range (lower weights overestimated and higher weights underestimated) for the positioning of both categorical boundaries and exemplars. Finally, the use of alternative weight status terminology resulted in an upward shift in the position of all boundaries in the BMI spectrum but failed to remove the compressive stimulus response effect. Discussion: There is a disconnect between the child size that people perceive to correspond to the different weight categories and the size criteria used by health professionals, and it is likely that this gap can only be bridged by training to recognise the medically based categories.

## 1. Introduction

Being overweight or obese during childhood is a major issue for public health, impacting an increasing number of children [1,2]. Being overweight in childhood predicts an array of negative psychosocial and physical problems over a person’s lifetime as weight status tends to persist over time [3]. The risks include cardiovascular disease, some cancers, type 2 diabetes [4], anxiety, depression, and low self-esteem [5]. School and family-based behavioural interventions can be used to ameliorate this problem [6,7], and there are a range of behaviour change techniques available to assist families [8]. However, intervention uptake depends on detecting that there is a problem, and parents consistently underestimate their child’s weight status [9].

### 1.1. Misperception of Child Body Weight: Extant Research and Theory

Previous studies show that parents usually judge their child’s weight to be in the middle of the BMI range (the “healthy” weight) even when it is objectively higher or lower according to age- and sex-standardised growth reference values [9,10,11]. This pattern of categorisation has been blamed on lower levels of parental education and health literacy, higher parental BMI, and child/parent gender and age [12,13]. Alternatively, children’s higher weight may be undetected because it is assessed by comparison to body size norms, and the general population now contains a higher proportion of larger bodies [14,15]. It is argued that this increase in the average BMI may cause a recalibration and upward shift in the perceptual threshold for when a child appears overweight [14,16]. In this study, we explore other alternative explanations.

Contraction bias represents one such alternative [17]. This is a visual bias that is a normal feature of our perceptual systems, and it affects how we estimate magnitude. In the case of body size estimation, contraction bias presupposes that the body being judged is compared to an internal reference body. This reference body is based on all the bodies one has seen previously. The most accurate size estimations happen when the size of the body being judged is close to the size of the reference body. However, as the magnitude of the difference between the body being judged and the reference body increases, so does the error in the estimation of size. As a result, bodies smaller than the reference average are judged as larger than they actually are, while bodies larger than the reference average are judged as smaller (see, e.g., [18,19,20,21,22,23,24]). This leads to a narrowing or “compression” of the range of BMI sizes estimated by observers (i.e., participants are less likely to use the extreme ends of a measurement scale). As a result of contraction bias, some bodies which fall outside the objectively defined healthy weight range will be perceived to fall within this category (the heavier bodies are underestimated, and the lighter bodies are overestimated). Critically, however, contraction bias can only be formally detected if the units of the response, e.g., estimated own body, are the same units as those of the body size of the stimuli used (e.g., BMI units).

#### 1.1.1. Linguistic Influence

Other potential cognitive reasons for the tendency of parents to underestimate their child’s weight status include the idea that parents either misapply eight categories or deliberately avoid them [25]. Terms such as “overweight” or “obese” are widely considered stigmatising [26], and some evidence suggests that parents try to avoid applying them to their child [27]. This could extend to a reluctance to apply the terms to children more generally [23]. Consequently, parents may be able to accurately estimate the shape and size of their child’s body but may not wish to categorise them with a negative weight descriptor. For example, terms such as overweight or very overweight might be regarded as pejorative, and parents may be reluctant to apply them to images of children. This may partly explain the miscategorisation reported by previous research [9,10,11,28]. If true, it suggests that the substitution of less contentious terms might improve the accuracy of weight status judgements, and their use by healthcare professionals (HCPs) might be of aid in consultations with parents about their child’s weight. Therefore, in this study, we also compared participants’ ability to define weight category boundaries using the standard set of body weight descriptors defined by the National Child Measurement Programme (NCMP) against terminology related to parents’ preparedness to act on behalf of their children’s health, and which is arguably less pejorative (i.e., “positive action”).

#### 1.1.2. Attitudinal Influences

Individuals who endorse specific causes for child weight tend to vary in their readiness to use potentially stigmatising labels. For example, Joslyn and Haider-Markel [29] reported that members of the public who believed in a genetic explanation for weight were more sympathetic to individuals who were overweight and resisted the use of stigmatising and discriminatory practices and policies. Consistent with this result, additional studies have reported that beliefs about the extent to which a person is responsible for their weight due to controllable factors (dietary intake and physical activity) are correlated with negative and stigmatising views on higher weight [30,31,32]. By contrast, ascribing weight gain to external and/or biological causes is not associated with such stigmatization [30,31,32]. Consequently, people who emphasise the role of factors such as genetics might be expected to be less willing to assign potentially stigmatising labels to child weight. We therefore tested this by including covariates which allowed us to examine relationships between participants’ assignment of weight boundary locations and their causal attributions for child weight.

### 1.2. Measurement Method Limitations

Previous studies which measured judgements of children’s weight status have used figural scales consisting of a series of figures depicting weight change. However, body scales are not a tool well suited for locating where participants believe categorical boundaries lie. They tend to employ a comparatively low number of body stimuli to represent BMI change, usually between seven and nine bodies (see [33]). This coarse sampling of the BMI range means that it is not possible to pinpoint the location in the BMI range where someone believes that one weight status category ends and the next begins with great accuracy and precision. To address this problem in the current study, we use a method of adjustment (MoA) task, where the BMI of the body being viewed can be altered in one-BMI centile steps by the participant to select a body shape/size that matches the task requirements. This finer sampling of the BMI range allows a more accurate and precise localisation of the categorical boundaries with one-BMI centile resolution.

### 1.3. Design Constraints

A common feature of many studies is the use of within-participant designs. For example, female participants might be asked to estimate their current body size, their ideal body size, and the body size they believe would be most attractive to men. For a comparison of the relationship between these three judgements, particularly the difference between their current and ideal bodies which is often taken as an operational definition of body dissatisfaction, there is no obvious alternative to this design. However, within-participant designs also have clear disadvantages. For example, asymmetric transfer from “companion” conditions may influence participant responses. Asymmetric transfer is defined as a strategy which a participant develops in one condition and which they then carry over and use in a subsequent condition where it is inappropriate. It has greatest impact when two or more conditions require different strategies, when the strategies are unobtrusive, and when the conditions are randomly interleaved in a block of trials [17]. For this reason, we have used a between-participant design where three separate groups of participants were randomly allocated to the different conditions.

### 1.4. The Current Study

The current study addresses two questions: (1) What body sizes/shapes do participants believe correspond to the NCMP-defined weight categories, and where do they place the boundaries between categories? (2) Is accuracy at this task improved by using terminology aimed at encouraging positive action by parents, as opposed to NCMP terminology, which is potentially more pejorative? “Positive action terminology” here is reflected in responses to questions such as “What is the smallest BMI a child can have before you would consider them unhealthy?” (see Supplementary Materials for further details).

To do this, we used photorealistic computer-generated imagery (CGI) stimuli based on 3D scans of child volunteers in an MoA task which allowed participants to select body sizes/shapes that corresponded to the task demands. To address the first question, we asked one set of participants to estimate the boundary positions between weight status categories as described by the UK National Health Service (NHS) and the NCMP terminology for children aged 4–5 and 10–11 years old. As a test of the validity of these judgements, we asked a second set of participants to use the MoA task to illustrate body sizes/shapes that they believed represented exemplars of each weight category using the NCMP terminology. We first predicted that the size of these exemplars should fall midway between the corresponding estimated boundaries (e.g., the estimated BMI of a healthy weight exemplar should fall midway between the estimated underweight–healthy weight and healthy weight–overweight boundaries).

Given that previous studies have suggested that people underestimate the weight status of overweight children and overestimate the weight status of underweight children [9,10,11], we also predicted that our participants would choose weight status boundaries that are higher in the BMI centile range than the NCMP boundaries for the overweight and very overweight categories, and lower in the BMI range relative to the NCMP underweight boundary. These differences in boundary position would produce the pattern of misestimations in child weight status previously reported.

To address the second question, we recruited a third set of participants to determine whether the wording used to refer to a child’s weight influenced the positioning of the boundaries. For example, instead of asking participants to move the MoA slider to the boundary between underweight and healthy weight (i.e., NCMP terminology), we asked them to find the body that they thought was the minimum BMI the child could be before the participant would consider them unhealthy. We predicted that using language which is more focused on positive action should encourage boundary selections which are closer to the official definitions.

Finally, we further predicted that participants would be less like to classify a body as overweight if they reported lower levels of child-focused weight stigma and supported genetic causes of being overweight.

## 2. Methods

### 2.1. Ethics and Pre-Registration

The Department of Psychology ethical committee at Northumbria University granted ethical approval for this study (Ethics references 35746 (pilot raw stimuli) and 4922 (main Daz stimuli)). We also pre-registered both the pilot and main study with the Open Science Framework (OSF): https://osf.io/xtq46 (pilot raw stimuli) and https://osf.io/w7rfb (main Daz stimuli) (both accessed on 16 May 2025).

### 2.2. Sample Size

We wanted to calculate appropriate participant sample sizes to estimate the critical relationship between BMI centile at each boundary location (i.e., UW/HW: underweight to healthy weight; HW/OW: healthy weight to overweight; and OW/vOW: overweight to very overweight) and the descriptor of the boundary itself (i.e., NCMP versus positive action). We also wanted to be able to test reliably for an interaction between these two factors, and therefore we required pilot data with which to estimate the sample size required for robust estimation of this interaction.

To do this, we first ran a pilot experiment using the MoA paradigm and the basic 3D body shapes produced by the principal component analysis of the child 3D scan data [28]. We recruited 45 white participants (42 female) into the NCMP judgement condition. The mean self-reported ages and BMIs for these participants were as follows: female age *M* = 21.45, *SD* = 5.68; male age *M* = 23.33, *SD* = 4.16; female BMI *M* = 24.50, *SD* = 5.76; male BMI *M* = 21.06, *SD* = 1.11. We also recruited 42 participants (35 female) into the positive action condition. The mean self-reported ages and BMIs for these participants were as follows: female age *M* = 24.46, *SD* = 8.77; male age *M* = 25.86, *SD* = 13.89; female BMI *M* = 22.91, *SD* = 3.95; male BMI *M* = 22.41, *SD* = 4.69.

We computed the means and SDs for estimated BMI centile, separately for the NCMP and positive action groups, split further according to boundary level (i.e., UW/HW, HW/OW, and OW/vOW) and stimulus type (i.e., older boys, younger boys, older girls, and younger girls). We also calculated the degree of covariation in estimated BMI centile separately as a function of boundary level and stimulus type. Together we used this information in GLIMMPSE [34] to calculate the sample size appropriate for estimating the interaction for the between-groups factor (i.e., judgement type: NCMP versus positive action), and the within-groups repeated measure (i.e., boundary location: UW/HW, HW/OW, and OW/vOW). At required powers of 0.8 and 0.9 and at alpha = 0.05, the respective sample sizes per group were 33 and 43.

### 2.3. Participants

Purposive sampling was used for the main study to recruit 128 White British parents of 4–5- or 10–11-year-old children on Prolific. Participants needed to be over 18, White British, a parent of a 4–5- or 10–11-year-old, understand written English, and not currently or previously diagnosed with an eating disorder. The ethnicity requirement was included as the bodies were rendered in Daz Studio to resemble White British children, and research suggests there are ethnic differences both in the perceptions of childhood obesity [35] and the pattern of fat deposition on the body [36].

### 2.4. Psychometric Measures

#### 2.4.1. Weight-Related Causal Attributions

Participants used eight separate visual analogue scales (VASs) to indicate the extent to which they believe eight factors contribute to higher weight in children (0 = not at all to 100 = completely). The list of factors was derived from Sahoo et al. (2015) [2] and included genetics, overeating, sedentary lifestyle, psychological factors (e.g., anxiety and depression), parental feeding style, fast food consumption, promotion of junk food, and sugary food consumption. The first three items in this list (genetics, overeating, and sedentary lifestyle) have been used previously when evaluating parental perceptions of the causes of higher weight in children [37]. In a validation sample of 202 participants, these items were significantly associated with scores on the “responsibility” subscale of the Fat Attitudes Assessment Toolkit (FAAT; [38]), which measures internal attributions for higher weight, in the expected directions (Pearson’s *r*(202) for genetics: −0.32; diet: 0.38; physical activity: 0.25; all *p* < 0.001). Over a period of 7 days, test–retest reliability was found to be adequate for each VAS item (Pearson’s *r*(101) for genetics: 0.76; diet: 0.64; physical activity: 0.60; all *p* < 0.001). The additional five factors were also included in this study to explore child weight-related causal attributions more comprehensively.

#### 2.4.2. Child-Focused Weight Stigma

A newly developed and validated child-focused weight stigma scale was included. This scale was developed due to the lack of existing questionnaires to measure the extent to which adults stigmatise children of higher weights [28]. This is a 15-item questionnaire that was developed from the Anti-Fat Attitude Test (AFAT; [39]) and changed references from *people* to *children* and made minor amendments throughout to ensure it made sense in the context of childhood. A 5-point Likert scale was used, from 1 = strongly disagree to 5 = strongly agree. Example items included “*Fat children have no willpower*”, and “*Most fat children are lazy*”. This child weight stigma scale has demonstrated excellent internal consistency (α = 0.90) and good test–retest reliability at the 7-day follow-up (*r* = 0.87). In this sample, Cronbach’s alpha was 0.88.

### 2.5. Psychophysical Measures

#### 2.5.1. Stimuli

The stimuli used in this study were created using 3D surface body scanning technology that captured accurate representations of 388 children aged 4–5 and 10–11-years-old to allow 3D Body Image Scales (BISs) to be created (see [19,40,41]). They were rendered in Daz studio, a 3D modelling software package, which resulted in them looking photorealistic (Daz Studio 4.10 from www.daz3d.com). This method is beneficial as it allows the same identity for the body in each of the stimulus sets to be maintained across a wide BMI range. The Daz stimuli improved those of Jones et al. [42] by incorporating advances in the realism with which CGI body stimuli are created, and details of their creation are given in Ridley et al. [43].

The age groups chosen are those monitored by the NCMP in English schools as part of the national paediatric weight surveillance programme [37]. Each set of stimuli was presented in an MoA task hosted by Pavlovia.org, which requires participants to move their mouse horizontally within a designated space to smoothly change the BMI centile (BMIc) of the BIS in one-centile steps. At the start of each trial, the BMIc of the body was set randomly, and the participant moved their mouse left within the green bar to reduce the BMIc to a minimum of the 1st centile, and right to increase the BMIc to the maximum of the 100th centile. To avoid participants remembering where they pressed in the previous trial, the space in which the participants could move their mouse was shifted on each trial (see below).

#### 2.5.2. MoA Task

There were three conditions to this MoA task, with participants assigned randomly to one of the conditions. The NCMP boundaries condition asked participants to move the mouse to find a child’s body that is on the boundary between (a) an underweight body and a healthy weight body, (b) a healthy weight body and an overweight body, and (c) an overweight body and an extremely overweight body. In the positive action condition, rather than using the NCMP terminology, they were given descriptors about when a parent should intervene to help control their child’s weight. Participants were asked to move the mouse to find a child’s body that was (a) the minimum BMI they could be before you would consider them unhealthy, (b) the maximum BMI they could be before you would consider them unhealthy, and (c) where you thought increasing BMI meant that the child had become extremely unhealthy and their parent should act urgently (e.g., encourage healthy eating and physical activity). The positive action labels were created using guidance from previous research that suggests that terminology such as “unhealthy weight” and “BMI” are more person-centred and less stigmatising and offensive [26,44,45]. Finally, in the NCMP exemplar condition, participants were required to decide where along the continuum of child bodies they believed the child body was a good example of underweight, healthy weight, overweight, and extremely overweight.

**Figure 1:**
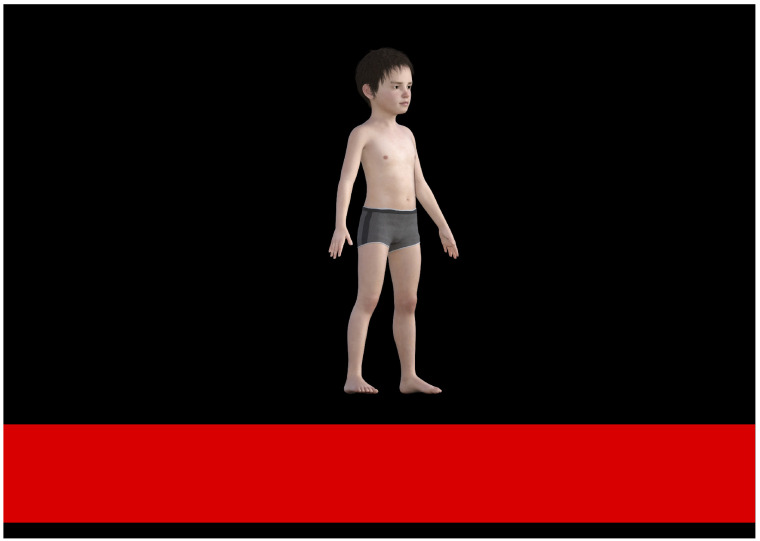
Screen shot of the arrangement of the avatar stimulus and response region on each trial of the MoA task immediately after participants clicked on the white cross. In this example, the image is of a young boy with a BMI centile of 50. See text for details.

Each trial of the MoA task began with the participant clicking a central white plus sign on a black screen to turn it green. This action triggered the appearance of a child avatar scaled to 70% of the device’s screen height while maintaining the original image aspect ratio. The initial appearance of the avatar was randomised between its lowest and highest BMI settings from one trial to the next. Below the stimulus image appeared a horizontal red rectangle which stretched to the full width of the screen. This is illustrated in Figure 1.

Participants were asked to move the mouse cursor into the red zone, which immediately turned green, and caused the mouse cursor to become invisible. Once in the green zone, they were encouraged to move the mouse leftward to reduce the body size of the image and rightward to increase its body size. In this way, participants used horizontal mouse movements to find a body size/shape that they believed best represented the task demands and clicked the left mouse button to register their choice. This action triggered the reappearance of the white plus sign in the middle of a black screen and initiated the next trial. Pilot testing showed that this arrangement removed any spatial cues that participants might otherwise rely on to remember where to move the mouse to from one trial to the next and forced them to focus only on body size change caused by their horizontal mouse movements.

Participants completed the task for all four stimulus sets of child bodies, and each label was trialled five times. In total, participants in the NCMP exemplar condition made 80 judgments using the MoA task (4 child groups × 4 labels × 5 repeats of each label). Those participants in the NCMP and positive action boundaries conditions completed 60 trials, given there were only three sets of instructions in these conditions rather than four. The order of the presentation of the blocks was randomised.

### 2.6. Procedure

Upon clicking the link to Qualtrics, participants were briefed in detail about the study, which provided enough information to allow them to give informed consent. The introductory information makes it clear that the experiment will involve judgements of the weight status of child CGI bodies. It is also explained that participants can withdraw at any point and their data will be deleted. Once consent was given, participants had to then confirm that they were using an eligible device to complete the survey (desktop PC or laptop). Mobile phones and tablets could not be used, and those that declared they were using one of these ineligible devices were automatically directed to the end of survey; here, it explained that they did not meet the eligibility criteria to continue. If any participant claimed that they were using an eligible device, but were actually using a mobile phone or tablet, the software running on Pavlovia.org detected and recorded this, and these participants’ data were also excluded from the study. After this, demographic data were self-reported by the participants, which included their gender (man, woman, non-binary, prefer not to say, prefer to self-describe), age, ethnicity (to confirm they were White British), height (in centimetres or feet and inches), and their weight (in kilograms, or stones and pounds, or pounds). To allow us to exclude anyone who may have found completing psychological measures about body image, depression, and eating disorder symptomatology upsetting, participants were also required to confirm they did not have a current or previous diagnosis of an eating disorder. Next, participants were asked to confirm their education or professional status. Those participants who did not identify as being a student (undergraduate or postgraduate) were asked to describe their current occupation. Participants also recorded how many children under 18 they had, their age(s), and gender(s). Participants then rated the extent to which they believed the eight factors outlined by Sahoo et al. [2] cause childhood obesity on a visual analogue scale ranging from 0 (not at all) to 100 (completely).

Next, participants were directed to Pavlovia.org where they were randomly assigned to either the NCMP exemplars condition, the NCMP boundaries condition, or the positive action condition of the MoA task (see Supplementary Materials for the validation of the terminology used in the positive action condition). Upon completion of the task, participants were redirected back to Qualtrics, where they completed the Child-Focused Weight Stigma questionnaire. Before the study debrief, they were asked an additional question about how comfortable they were when applying the weight labels to the child stimuli in the preceding task on a scale from 0 to 100 (0 = very uncomfortable, 100 = very comfortable). They were also asked to what extent they believed child obesity (i.e., obesity in children aged 11 and under) is controllable by (a) the child and (b) their family (0 = very uncontrollable, 100 = completely controllable). These items have previously been utilised in research to determine attitudes towards child weight status and were shown to predict parents’ and HCPs’ accuracy in weight status categorisations [25]. On average, the study took 30 min to complete.

## 3. Results

The data files are available through the Open Science Framework (OSF) at https://osf.io/qe8km/ (accessed on 16 May 2025).

### 3.1. Univariate Statistics

We obtained complete datasets from 128 adults who carried out the MoA task with the Daz stimuli. Forty-seven (23 females, 24 males) were assigned to the positive action condition, 41 (26 females, 15 males) to the NCMP boundary condition, and 40 (17 females, 23 males) to the NCMP exemplar condition. Participant body mass index (BMI) was calculated (weight/height^2^) after converting self-reported weights to kilograms and heights to metres. Table 1 shows the characteristics of these participants.

Table 1 illustrates how participants’ ages fell in the range ~25 to ~55, and their BMIs between ~18 and ~45. We used PROC MULTTEST (SAS v9.4) to compute pairwise comparisons for all participant characteristics for the three groups of participants. These pairwise comparisons were controlled for multiple comparisons, and none were statistically significant at *p* < 0.05.

### 3.2. Multivariate Statistics: NCMP Boundaries and Exemplars

As a first step, we wanted to analyse the relationship between participants’ judgements of the locations of NCMP boundaries in BMI centile units (i.e., between underweight and healthy weight (UW/HW), healthy weight and overweight (HW/OW), and overweight and very overweight (OW/vOW)) and the actual BMI centile values defined by the NCMP for these boundaries.

To do this, we built a linear mixed effects (LME) model of the estimated boundary values, using PROC MIXED in SAS v9.4 (SAS Institute, Cary, NC, USA). The fixed effects we tested were boundary location (i.e., UW/HW, HW/OW, and OW/vOW), stimulus type (i.e., older boy, younger boy, older girl, or younger girl), and the two-way interaction between the two. In addition, owing to the asymmetry in the numbers of males and females in the study groups, we included observer sex as a covariate. We included a random effect for the influence of participant on intercepts. Degrees of freedom were estimated using the Satterthwaite method. For dummy-coded (reference-coded) variables, the control levels were OW/vOW for boundary location and younger girl for stimulus type. Effects were retained in the model if their Type III test of fixed effect was significant at *p* < 0.05 and they contributed to a substantial change in AICc [46]. Fixed effects which were non-significant in isolation but nevertheless contributed to significant interaction terms were also retained.

The variance in intercepts across participants was significant (*Z* = 4.39, *p* < 0.0001). We found, and retained, significant Type III tests of fixed effects for boundary location (*F*(2,946) = 738.41, *p* < 0.0001) and stimulus type (*F*(3,946) = 19.60, *p* < 0.0001). Neither the effect of observer sex, nor the interaction between boundary location and stimulus type were statistically significant. The final model explained 56% of the variance in estimated BMI centiles relative to the unexplained variance in estimated BMI centiles [47,48] with an effect size *f*^2^ = 1.26. Table 2 shows the least square mean (LS-mean) values for BMI centiles at each NCMP boundary location, separately for the four stimulus types.

It is clear from Table 2 that the LS-means for the estimated boundary locations are substantially higher for the UW/HW boundary and substantially lower for both the HW/OW and OW/vOW boundaries. Indeed, there is no overlap between the official NCMP boundary values and the 95% confidence intervals for the LS-means.

**Figure 2:**
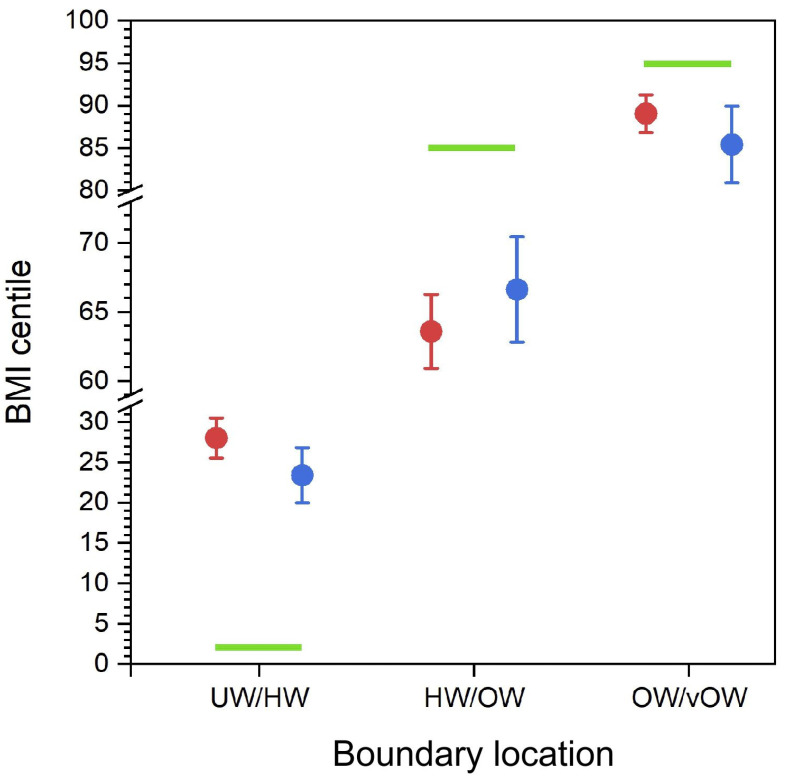
The BMI centile values of the boundary “guesses” (i.e., the means of UW and HW, HW and OW, and OW and vOW exemplars) are plotted in red, and the directly estimated boundary estimates are plotted in blue, as a function of the NCMP boundary location names. Error bars represent 95% confidence intervals. The green bars represent the upper limit for the UW/HW boundary at BMI centile 2%, the lower limit for the HW/OW boundary at BMI centile 85%, and the lower limit for the OW/vOW boundary at BMI centile 95%.

Finally, we wanted to check that the so-called boundary estimates provided by participants do indeed constitute boundaries. For this to be true, we would expect that, for example, the BMI centile for the UW/HW boundary is higher than the BMI centile for the UW exemplar, and lower than the BMI centile for the HW exemplar. To test this, we computed the mean BMI centiles with their 95% confidence intervals for the UW and HW exemplars, the HW and OW exemplars, and the OW and vOW exemplars. These averaged exemplar “guesses” for boundaries are plotted with their 95% CIs in red in Figure 2. In blue, we also plotted the mean BMI centiles for each of the UW/HW, HW/OW, and OW/vOW boundaries that participants judged directly, together with their 95% CIs. It is clear from Figure 2 that in each case the confidence intervals for the actual boundary estimates and the “guesses” based on exemplar estimates are overlapping for UW/HW, HW/OW, and OW/vOW locations.

### 3.3. Multivariate Statistics: Comparing NCMP and Positive Action Boundaries

In our second analysis, we wanted to ask whether using boundary descriptors that likely reflect a parent’s preparedness to act on behalf of a child’s health might produce boundary estimates that reflect the NCMP boundary definitions more accurately. To do this, we built another LME model using PROC MIXED in SAS. The fixed effects we tested were boundary location (i.e., UW/HW, HW/OW, and OW/vOW), condition (i.e., the language used to describe the boundary decision—positive action versus NCMP), as well as stimulus type (i.e., older boy, younger boy, older girl, or younger girl). We tested all possible two- and three-way interactions for the fixed effects. In addition, we wanted to test for the influence of the following covariates: participant age and BMI, the extent to which they attribute the eight factors outlined in the methods (e.g., genetic influences, overeating, and lack of physical activity) as causes of child overweight, and their comfort, controllability, and child-focused weight stigma scores. We included a random effect for the influence of participant on intercepts. Degrees of freedom were estimated using the Satterthwaite method. For dummy-coded variables, the control levels were OW/vOW for boundary location, positive action for condition, and younger girl for stimulus type. Effects were retained in the model if their Type III test of fixed effect was significant at *p* < 0.05 and they contributed to a substantial change in AICc [46]. Fixed effects which were non-significant in isolation, but nevertheless contributed to significant interaction terms, were also retained.

**Figure 3:**
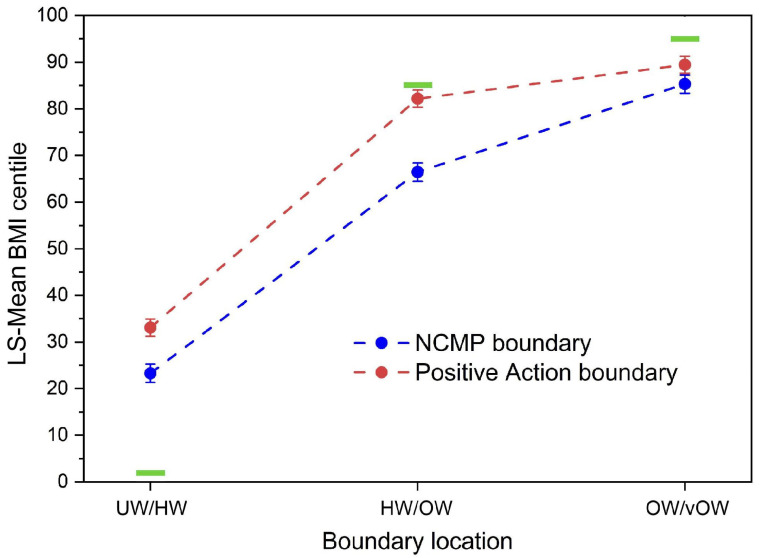
Scatter plot showing LS-Mean BMI centile plotted as a function of boundary location, separately for the NCMP (blue) and positive action (red) conditions. Error bars represent ±1 SEM. The green bars represent the upper limit for the UW/HW boundary at BMI centile 2%, the lower limit for the HW/OW boundary at BMI centile 91%, and the lower limit for the OW/vOW boundary at BMI centile 98%.

The variance in intercepts across participants was significant (*Z* = 4.72, *p* < 0.0001). We found, and retained, significant Type III tests of fixed effects for boundary location (*F*(2.939) = 1034.17, *p* < 0.0001), condition (*F*(1.83) = 20.83, *p* < 0.0001), stimulus type (*F*(3.939) = 8.85, *p* < 0.0001), and the interaction between boundary location and condition (*F*(2.939) = 8.98, *p* < 0.0001). The only covariate that contributed significantly to the model was psychological factors (*F*(1.83) = 4.21, *p* = 0.04). No other covariate made a significant contribution to the final model. This model explained 63% of the variance in estimated BMI centiles [47,48] with an effect size *f*^2^ = 1.70, and its parameters are reported in Table S1 in the Supplementary Materials. The model is illustrated by the scatter plot in Figure 3.

Table S1 and Figure 3 show that, averaged across stimulus type, the BMI centiles for boundary location increase systematically as a function of boundary location. The positive action boundaries are higher than those for the NCMP boundaries for UW/HW and HW/OW but converge towards each other for OW/vOW. Table S1 shows that, on average, the boundaries for older and younger girls are similar to each other, as are the boundaries for older and younger boys. However, on average, the boundaries are significantly higher by around 6 centile points for boys than girls. Finally, the effect of psychological factors (i.e., “To what extent do you think that child obesity is caused by psychological factors such as depression and anxiety”) is to systematically and significantly increase boundary estimates as it increases.

## 4. Discussion

This study firstly asked which body sizes our participants believed corresponded to the NCMP-defined weight categories, and where they placed the boundaries between categories. The results suggest that the perceived position of the boundaries in the BMI range is significantly different from the position of those corresponding to the NCMP categories and the weight status categories in the BMI centile scales produced by the Royal College of Paediatrics and Child Health (RCPCH) (https://growth.rcpch.ac.uk/clinician/growth-references (accessed on 16 May 2025)). The perceived overweight and very overweight boundaries are significantly shifted towards lower BMI centiles, and the underweight boundary is shifted towards higher BMI centile values (see Figure 2 and Figure 3). This leads to an apparent compression of the response range used by our participants. This is true of both the positioning of categorical boundaries and the position of the exemplars on the BMI spectrum. These results show internal consistency in the results (i.e., the positions of the exemplars in the BMI range fall roughly midway between the corresponding categorical boundaries). It is important to note that these judgements are not a judgement of the absolute size of a body, but instead the body size that corresponds to a particular weight category (i.e., a modified categorisation task, not a size estimation task).

We also tested whether these judgements were modulated by linguistic factors by using a set of alternative weight status descriptors. This alternative terminology emphasised positive action in response to weight status rather than simple weight categorisation. Our participants’ boundary judgements for HW/OW and OW/vOW were closer in position to the boundaries used by the NCMP when using this alternative terminology, but further away for UW/HW—essentially all boundaries were located at higher BMI centiles. What the manipulation of the terminology did *not* do was to relax the compressive stimulus–response effect. For this to happen, we would have needed to see a much steeper curve for the positive action condition than the NCMP condition in Figure 3.

### 4.1. The Miscategorisation of Body Size

There is no a priori reason why we would expect anyone in the general population to make weight status judgements in line with the values used by the NCMP. However, previous studies using figural scales have reported a tendency to misclassify overweight, very overweight, and underweight bodies as being healthy weight (i.e., the bodies are seen as more like the healthy weight bodies in the middle of the BMI range). One implication of this is that for a body to be judged as overweight/very overweight, it needs to be significantly larger than its corresponding NCMP weight category suggests, and to be judged as underweight, it needs to be considerably thinner than its NCMP weight category suggests.

In the previous studies which used figural body scales, a participant was asked to match their child’s body size to a body from a scale in which the bodies were depicted as ascending in BMI. The weight status of each of the bodies in the scale was known, and the task tested how accurately a participant could match a child to a body of the corresponding weight category. Under such conditions, there is a tendency to choose one of the central bodies in preference to those at the extreme ends, no matter the BMI of the body they are matching to (i.e., whether the body to be matched is under-, over-, or healthy weight, a participant tends to match it to one of the central bodies in the image set which are all healthy weight) [9,10,11]. This pattern of results has been interpreted as being due to an overestimation of underweight bodies and underestimation of overweight bodies but may simply be produced by a compression of the range of responses made by the participants around the central bodies in a scale when tested using this paradigm.

In the MoA task, participants judged the weight status boundaries to be closer to the centre of the scale than the positions of the boundaries used by the NCMP. This again may be a compression of the range of responses made by the participants around the central position of the scale. This in turn leads to an apparent miscategorisation. In this case, heavier bodies within what the NCMP would classify as the healthy range are categorised as overweight or very overweight, and lighter bodies within the healthy range are categorised as underweight. Thus, this miscategorisation may simply derive from compression of the range of BMI values made in this paradigm. This effect could be exacerbated by a participant’s expectation that the four weight status categories would cover roughly equal BMI ranges, rather than the very disparate sizes that they cover in practice. The healthy weight category covers most of the BMI centile range, with very narrow ranges for the other three categories. Future studies using this technique could minimise some of the potential participant biases by emphasising in the explanatory material at the start of the testing that the weight status categories cover very different widths of the BMI ranges.

### 4.2. Weight Status Versus Action Terms

A key question is whether the compression effect might be due to the discomfort caused by the NCMP terminology. If we assume that the alternative, positive action descriptors do not elicit the same discomfort triggered by the NCMP language (see Supplementary Materials for details of the positive action condition), then it appears that this is not the case. Although the change in terminology does alter the position of the weight categories, which is reflected in a higher intercept when BMI centile is plotted as a function of boundary location (see Figure 3), it does not change the gradient of the function towards a steeper value (i.e., there is no reduction in response compression). The weight category boundaries are simply all shifted upwards by ~10 to ~20 BMI centile units. The potential caveat here is that the action language elicits a different form of participant discomfort, which leads to an equivalent compressive effect, again driven by the language used to classify weight status, but nevertheless different from that produced by the NCMP language.

Additionally, the participants filled in a set of questions to index their beliefs about the causes of child weight (such as “To what extent do you think that child obesity is caused by psychological factors such as depression and anxiety”). These psychological factors significantly predict the position of the categorical boundaries. Higher psychological scores predict an increase in the BMI centile value of the boundary estimates. This is a general upward shift of all the boundaries (including the underweight boundary) towards higher BMI centile values. This demonstrates significant attitudinal factors (as opposed to primarily perceptual factors) in weight status judgements, with what an observer believes about the causes of weight gain modulating their judgements. Previous studies have suggested that beliefs about the causes of potential weight gain impact an observers’ judgements of weight status [25,28]. Where participants believed a child’s weight was due to factors that were not under their own control, observers were less likely to categorise them as being overweight (effectively shifting the boundaries to higher BMI levels). We find the same pattern in this study. Participants who believed that a child’s weight is at least partially due to psychological factors (which are beyond a child’s control), also chose categorical boundaries at higher BMI values.

### 4.3. Putative Mechanisms Underlying Response Compression

A potential explanation for this pattern of results is related to anchor effects, and a reluctance for participants to make use of the upper and lower limits of the response range. This effect is particularly common if, at the beginning of a trial, the starting point of the cursor is in the middle of the rating scale. In this case there may be a tendency to make judgements biased towards that central starting point [17]. To counter this issue, on each trial we positioned the cursor starting point at the very ends of the rating scale (either at the top or the bottom). This format should have encouraged participants to use the full range of the scale.

Additionally, the BMI range of stimuli used in this and future studies should be considered. We used bodies varying in BMI centile for each age and gender from 1 to 100. However, this range may have limited the choices made by our participants, and one option would be to extrapolate body size in the stimuli significantly beyond the 100th centile range. This would ensure that any choice of body size that an observer might like to make sits well within with stimulus range available. There is some precedent for this; for example, the BMI weight charts for children in the UK provide additional BMI trajectories for children at +3, +3.33, +3.66, and +4 SD above the 100th centile [49]. In previous studies on adult male body size preferences, a similar slider-based technique used a very wide range of size and shape change to minimise potential ceiling effects [50,51]. With a wider range of child body sizes, the range of weight status boundary choices made by participants might now expand to select both larger and smaller images for the respective overweight and underweight categories.

### 4.4. Limitations and Future Directions

An important limitation is that we are using stimuli based on only White children, and the judgements are made by White parents. There are significant differences in the amount of adipose tissue for a given BMI and its distribution on the body between different ethnic groups [52,53,54]. This potentially leads to different body shapes between different ethnic groups for a given BMI, and so to accurately represent these differences in figural scales, it will be necessary to 3D scan representative examples of children from different ethnic groups. While stimulus image development (scanning, data analysis, and rendering) is costly, this can be done in high- income countries with an ethnically diverse population. Then, usage of the stimuli in low-income countries can be achieved relatively cheaply through an app run on a tablet, thereby minimising cost implications. Moreover, the BMI cut-off values for the weight status categories differ in different ethnic groups, further complicating this picture [55]. Thus, we would strongly argue that to accurately test the perception of weight status in a modern multi-ethnic population, it is important to recruit volunteers from each ethnic group and use child stimuli created using biometric data from that specific group. Simply assuming that the judgements of one ethnic group will extend to the judgements made by other groups may be inaccurate, and using a mixed set of observers, combining participants from multiple ethnic groups who may use different criteria to make their judgements, may obscure differences in weight judgements. There may also be differences based on cultural differences which should be explored [56,57]. Additionally, we excluded people with a history of eating disorders from our participants, as previous studies have suggested significant differences in their accuracy of adult body size estimation relative to healthy controls [58,59]. Further studies should consider whether these differences extend to parental child judgements.

Our results suggest that peoples’ responses to categorising child weight status are very malleable. They can be altered both by the paradigm used to test their judgements (such as MoA versus figure rating scales) or by the phrasing used to frame the task (such as NCMP nomenclature versus positive action terms). Participants can judge the relative size of bodies but are poor at allocating them to the correct weight status category [25,28,43]. This suggests care must be taken in interpreting the results of such studies when trying to draw conclusions about the accuracy of people’s weight categorization, but also that people do not seem to have a very clear idea about what body size corresponds to the different weight categories. This is important because correctly recognising child weight status matters if a child becomes under- or overweight. Although interventions exist to help parents and children manage their weight, they will only be accessed if a problem is recognised. Thus, the development of aids to accurate recognition of child weight status are arguably essential for good public health.

### 4.5. Conclusions

Our results suggest that the perceived position of the weight status boundaries in the BMI centile range is significantly different from the position of those boundaries used by the NCMP. The position of these boundaries is modulated by the wording of the weight status description, consistent with a significant attitudinal impact on these judgements. Future studies should consider how the paradigm used to test weight status judgements can influence their results and take the appropriate steps to minimise this effect.

## Figures and Tables

**Table 1:** Participant characteristics.

	NCMP Boundary (*n* = 41)		NCMP Exemplar (*n* = 40)		Positive Action (*n* = 47)	
	M	SD	M	SD	M	SD
Age (years)	36.98	6.42	40.78	5.77	37.94	6.97
BMI (kg/m^2^)	27.95	4.25	26.92	5.04	27.81	6.40
Comfort_Child (0–100)	55.29	32.57	55.53	33.43	53.53	35.87
Control_Child (0–100)	29.51	21.47	29.23	19.81	25.19	19.45
Control_Family (0–100)	71.44	18.67	79.25	17.03	77.45	19.54
Genetic (0–100)	30.02	19.18	35.35	21.75	34.30	20.15
Parental Feeding Style (0–100)	65.78	22.13	73.60	19.45	71.85	20.41
Psychological Factors (0–100)	43.12	22.63	42.00	25.10	42.70	24.79
Overeating (0–100)	76.56	18.31	82.23	14.85	79.87	14.54
Sedentary Lifestyle (0–100)	67.39	23.56	77.93	20.09	74.11	18.02
Fast Food Consumption (0–100)	61.71	20.9	67.85	24.51	68.00	23.13
Promotion of Junk Food (0–100)	45.07	20.75	46.13	25.04	42.72	26.29
Sugary Drinks Consumption (0–100)	56.49	20.62	59.40	27.00	55.09	26.79
CFWS (1–75)	46.37	8.59	47.78	9.91	48.17	10.69

*Note*: BMI = Body Mass Index; CFWS = Child-Focused Weight Stigma.

**Table 2:** LS-mean values for BMI centiles at each NCMP boundary location.

	Boundary	NCMP Boundary	Boundary	
Stimulus	Name	Values	LS-Mean	95% CI
Older Boys	UW/HW	2	26.09	22.01–30.16
	HW/OW	85	69.60	65.53–73.67
	OW/vOW	95	85.24	81.17–89.31
Younger Boys	UW/HW	2	22.81	18.74–26.88
	HW/OW	85	66.34	62.27–70.41
	OW/vOW	95	83.41	79.34–87.48
Older Girls	UW/HW	2	26.19	22.12–30.26
	HW/OW	85	62.27	58.20–66.34
	OW/vOW	95	82.28	78.21–86.35
Younger Girls	UW/HW	2	19.03	14.95–23.10
	HW/OW	85	52.49	48.42–56.56
	OW/vOW	95	75.20	71.13–79.28

*Note*: UW = Underweight; HW = Healthy Weight; OW = Overweight; vOW = Very Overweight.

## Data Availability

The data files are available through the Open Science Framework (OSF) at https://osf.io/qe8km/ (accessed on 16 May 2025).

## Supplementary Materials

The following supporting information can be downloaded at https://www.mdpi.com/article/10.3390/children12070836/s1: This provides details of development of the Positive Action terminology and Table S1 provides details of the linear mixed effects model parameters from estimated BMI centile data for the Boundary study.
