# Supplementary material for: Can Adults Accurately Judge Child Weight Status?
Source: Children (Basel). 2025 Jun 25;12(7):836. doi: 10.3390/children12070836 (PMC12293906; doi:10.3390/children12070836)
Supplement: Supplementary file 1 [file children-12-00836-s001.zip › children-3667209-supplementary.pdf]

Supplementary Materials

1: Development of the Positive Action Terminology

Before carrying out the main study, we ran an initial study to evaluate the terminology we would be using in the positive action condition, and to demonstrate that the terminology is more likely to lead to parents' taking action to improve their children's health. We recruited 70 participants via opportunity sampling using social media and [Blanked University] SONA system. They first self-reported their gender (man, woman, non-binary, prefer not to say, prefer to self-describe), age, ethnicity, height (in centimetres, or feet and inches) and weight (in kilograms, or stones and pounds, or pounds). They then provided their student or professional status, and confirmed whether they had any children under 18, and if so, how many and what gender(s).

Next, participants were presented with both the NCMP boundary and positive action labels for each of the three boundary levels and asked to choose which of the two descriptors they thought would be most likely to lead to useful action by the parent, on behalf of a child. They did four repeats of this task before being debriefed. This study took no longer than 10 minutes.

Terminology Used

The NCMP terminology used the phrasing "the boundary between a) an underweight body and a healthy weight body, b) a healthy weight body and an overweight body and c) an overweight body and an extremely weight body."

Quantitatively, the full set of NCMP boundaries are as follows:

BMI centile <=2: Underweight

BMI centile >2 and <85: Healthy weight

BMI centile >=85 and <95: Overweight

BMI centile >=95 (between 95 and 100): Obesity

BMI centile >=99.6 (between 99.6 and 100): Severe obesity. Note: this is a subset of "Obesity"

(Please see: https://digital.nhs.uk/data-and-information/publications/statistical/national-child-measurement-programme/2023-24-school-year/introduction#:~:text=to%20local%20level.-,Definitions%20used,is%20a%20subset%20of%20%E2%80%9CObesity%E2%80%9D)

For the current study, we have concatenated Obesity and Severe obesity to create vOW. Thus, in the current paper: 0 < UW <= 2 BMI centile units; 2 < HW <= 85 BMI centile units; 85 < OW <= 95 BMI centile units; 95 < vOW BMI centile units.

In the positive action terminology, participants were given descriptors about when a parent should intervene to help control their child's weight: "The child's body that was: a) the minimum BMI they could be before you would consider them unhealthy, b) the maximum BMI they could be before you would consider them unhealthy and c) where you think increasing BMI means that the child has become extremely unhealthy, and their parent should act urgently (e.g. encourage healthy eating and physical activity). "

The positive action labels were created using guidance from previous research that suggests that terminology such as "unhealthy weight" and "BMI" are more person-centred and less stigmatising and offensive (Auckburally et al., 2021; Fisch et al. 2021; Puhl, 2020).

The distinction between NCMP versus Positive Action labels

We recruited 70 white participants (57 females) to judge which of these two labels was most likely to lead to parents' seeking to improve their children' health. The mean self-reported ages and BMIs for these participants were: female age M=24.94, SD=9.92, male age M=26.08, SD=12.45, female BMI M=23.66, SD=4.32, male BMI M=24.51, SD=3.16. All but one participant self-reported currently living in the UK. Five of these individuals had children under the age of 18. Fifty-four self-reported being current undergraduate students, 9 as postgraduate students, and 7 as working in full time employment.

For each of the three weight boundary conditions, each participant was asked whether they would choose the NCMP descriptor or the positive action descriptor as the descriptor most likely to lead to action leading to improvements in children's health. The respective percentages for NCMP versus Positive action choices for the three boundary categories were: lowest boundary, 39.86% versus 60.14%; middle boundary, 39.86% versus 60.14%; highest boundary, 38.51% versus 61.49%. Chi-square tests for equal proportions (i.e., testing against NCMP choice 50% versus Positive action choice 50%) showed that in each case, participants were significantly more likely to favour the positive action choice over the NCMP choice (low boundary, $p = .0005$; middle boundary, $p = .0005$, highest boundary, $p < .0001$).

2: Linear Mixed Effects modelling parameters

Table S1. *Linear mixed effects model parameters from estimated BMI centile data for the Boundary study*

Effect	Condition		Boundary Location	Estimate	SE	t value (DF)	p value
Intercept				86.18	2.09	41.28 (288)	<.0001
Boundary location			UW/HW	-56.36	1.87	-30.21 (939)	<.0001
Boundary location			HW/OW	-7.27	1.87	-3.90 (939)	.0001
Boundary location			OW/vOW	0.00	.	.	.
Condition	NCMP Boundary			-4.10	2.71	-1.51 (187)	.1
Condition	Pos Act Boundary			0.00	.	.	.
Boundary location × Condition	NCMP Boundary		UW/HW	-5.67	2.74	-2.07 (939)	.04
Boundary location × Condition	Pos Act Boundary		UW/HW	0.00	.	.	.
Boundary location × Condition	NCMP Boundary		HW/OW	-11.59	2.74	-4.24 (939)	<.0001
Boundary location × Condition	Pos Act Boundary		HW/OW	0.00	.	.	.
Boundary location × Condition	NCMP Boundary		OW/vOW	0.00	.	.	.
Boundary location × Condition	Pos Act Boundary		OW/vOW	0.00	.	.	.
Stimulus type		BO		6.15	1.58	3.90 (939)	.0001
Stimulus type		BY		6.04	1.58	3.84 (939)	.0001
Stimulus type		GO		0.76	1.58	0.48 (939)	.6
Stimulus type		GY		0.00	.	.	.
Psychological Factors				0.093	. 0.045	2.05 (83).	.04.

Note. UW = Underweight; HW = Healthy Weight; OW = Overweight; vOW = Very Overweight; BO = Older Boys; BY = younger Boys; GO = Older Girls; GY = Younger Girls.
